# Biodegradable materials for bone defect repair

**DOI:** 10.1186/s40779-020-00280-6

**Published:** 2020-11-10

**Authors:** Shuai Wei, Jian-Xiong Ma, Lai Xu, Xiao-Song Gu, Xin-Long Ma

**Affiliations:** 1 Tianjin Hospital, Tianjin University, No. 406 Jiefang South Road, Tianjin, 300211 China; 2 Jiangsu Clinical Medicine Center of Tissue Engineering and Nerve Injury Repair, Key Laboratory of Neuroregeneration of Jiangsu and Ministry of Education, Nantong University, No. 19 Qixiu Road, Chongchuan District, Nantong, 226001 China

**Keywords:** Biodegradable materials, Bone defects, Bone repair, Intelligent material, Modular fabrication

## Abstract

Compared with non-degradable materials, biodegradable biomaterials play an increasingly important role in the repairing of severe bone defects, and have attracted extensive attention from researchers. In the treatment of bone defects, scaffolds made of biodegradable materials can provide a crawling bridge for new bone tissue in the gap and a platform for cells and growth factors to play a physiological role, which will eventually be degraded and absorbed in the body and be replaced by the new bone tissue. Traditional biodegradable materials include polymers, ceramics and metals, which have been used in bone defect repairing for many years. Although these materials have more or fewer shortcomings, they are still the cornerstone of our development of a new generation of degradable materials. With the rapid development of modern science and technology, in the twenty-first century, more and more kinds of new biodegradable materials emerge in endlessly, such as new intelligent micro-nano materials and cell-based products. At the same time, there are many new fabrication technologies of improving biodegradable materials, such as modular fabrication, 3D and 4D printing, interface reinforcement and nanotechnology. This review will introduce various kinds of biodegradable materials commonly used in bone defect repairing, especially the newly emerging materials and their fabrication technology in recent years, and look forward to the future research direction, hoping to provide researchers in the field with some inspiration and reference.

## Background

**Fig. 1:**
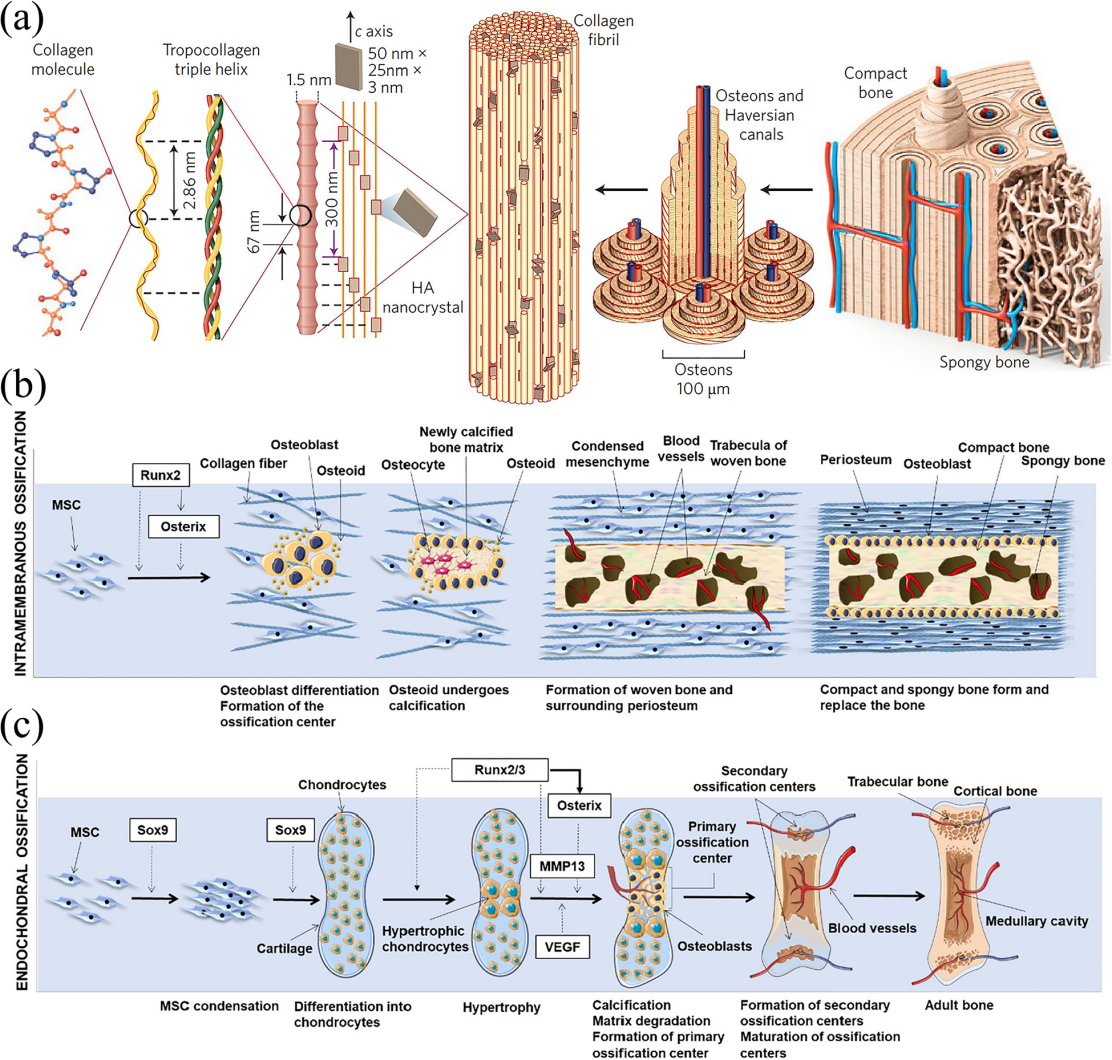
The hierarchical structure and healing mechanism of human bone. **a** The hierarchical structure and main nanostructure of human bone. The macrostructure of bone consists of spongy bone and compact bone, with bone and Haversian canals around blood vessels. At the micro level, bone tissue is mainly a three-dimensional (3D) nanostructure composed of nanohydroxyapatite and self-assembled collagen fibres. Reprinted with permission from [1], published by Springer Nature. b. Schematic representation of IMO, which mainly includes the following stages: differentiation of mesenchymal stem cells (MSCs) into osteoblasts with the participation of Runx2 or osterix, formation of the ossification centre, osteoid calcification, formation of woven bone and surrounding periosteum, formation of compact and spongy bone and replacement of woven bone. c. Schematic representation of EO, which is mainly composed of the following stages: condensation and differentiation of MSCs into chondrocytes with the participation of Sox9, hypertrophy, calcification, matrix degradation, primary ossification centre formation, secondary ossification centre formation, ossification centre maturation and adult bone formation. **b** and **c** are adapted by permission from [2], published by Elsevier

Bone is mainly composed of three components: cells, fibres, and matrix. The main component of the bone matrix is collagen, which provides tensile strength. The mineral component of bone is mainly calcium phosphate, which provides compressive strength (Fig. 1a) [1]. Its most notable feature is that the intercellular substance deposited contains a large quantity of calcium salts, which become a very hard tissue that forms the skeletal system of the body and provides support and protection for various organs [3]. There are many causes of bone defects/bone loss, such as trauma, orthopaedic surgery, osteoarthritis, osteoporosis, and primary tumour resection [4, 5].

There are several regenerable tissues/organs in the human body, including skin, liver, glands, blood, and especially bone. Generally, for minor injuries or small bone defects, the body’s bone tissue can be regenerated [6]. In the case of severe bone injury (critical-size bone defects or more serious injuries), natural bone grafts or biomaterials must be used to bridge the gap before bone regeneration can be achieved. However, the organism is a complex system, and this application is not as simple as it may seem. To repair bone defects more efficiently, we must consider the anatomical location of the bone defect, the blood flow, the degree of damage to surrounding tissues, whether there is a serious infection, the state of the body, and whether the defect is combined with metabolic diseases [7]. According to statistical estimates, approximately 20 million patients worldwide lose bone tissue due to various diseases every year [8]. At present, autogenous bone transplantation, usually taken from the patient’s own iliac bone, is still the gold standard for repairing serious bone defects. The obtained fresh autologous bone has unparalleled advantages over other grafts, including good histocompatibility, non-immunogenicity, an abundance of autologous progenitor cells, and good osteoconductivity [9]. However, there are also many shortcomings in autologous bone transplantation, including the limited amount of bone available and severe complications, such as donor-site haematoma, deep infection, inflammation, and prolonged hospital stay [10]. Allogeneic bone transplantation, usually taken from other patients or human bodies, can compensate for the lack of autologous bone mass to a certain extent, provide some growth factors and exhibit osteoinductive properties, which can actively induce new bone formation by activating the signalling pathways for bone regeneration and bone progenitor cell recruitment [11]. Unfortunately, these donor bone tissues carry the risk for recipient infection, disease transmission, and immune responses [10]. With the development of chemical and tissue engineering technologies, artificially modified bone xenograft materials have attracted great interest from researchers, which is usually obtained from mammals, such as pigs [12]. However, due to the potential risk of disease or virus transmission, infection, and immunogenicity, among others, some researchers do not recommend these materials for wide use in bone defect repair [13].

Due to the urgent need for the clinical development of bone repair materials that have the same structure and function as natural bone but are also non-immunogenic, bone tissue engineering has emerged and achieved rapid development in the past decade [14]. With the advantages of wide sources, adjustable parameters (personalized treatment), and no risk of disease transmission, synthetic materials are favoured by researchers. The first generation of bone graft substitutes consisted of bioinert materials, which have the common disadvantage of forming fibrous tissue at the interface, preventing the host tissue from fully integrating with the materials [15]. Despite their shortcomings, patients’ quality of life improved for 5 to 25 years after the implantation of an “inert” biomaterial. To improve tissue growth into bone graft materials, researchers have designed and developed second-generation bioactive materials. The concept of bioactivity refers to chemical bonding induced at the interface between materials and biological tissues, which was proposed by professor Hench in a study on bioglass in 1969, leading to the introduction of bioceramics [16]. Bone tissue engineering has developed into a highly active field in the past few decades that integrates knowledge and technology from different disciplines and is the most promising method for developing new third-generation bone graft materials. Tissue engineering-based bone defect repair scaffolds should be biocompatible, biodegradable, and osteoconductive with low immunogenicity [17]. At the same time, the bone tissue engineering strategy emphasizes inoculating the scaffold with cells or loading the scaffold with growth factors to achieve a slow-release effect, simulate the microenvironment of tissue regeneration in the body and accelerate the quality and speed of tissue regeneration [16]. In the past 20 years, with the rapid development of micro/nanotechnology and computer technology, new intelligent micro/nanomaterials have gradually come into being, which emphasizes the integration of nanotechnology, advanced biological materials and molecular biotechnology [18]. New functional intelligent materials can respond in a predetermined and predictable way according to specific environmental stimuli, including ionic strength, temperature, pH, thermokinetic compatibility of solvents, specific molecular recognition and other physiological signals [18, 19].

In the treatment of bone defects, scaffolds play an important role and can provide both a bridge for new bone tissue growth into the gap and a platform for cells and growth factors to play a physiological role [20]. Based on these characteristics of biocompatibility, osteoconductivity, low immunogenicity, and non-infectivity, we particularly emphasize the biodegradability of these materials, such as chitosan, poly (lactic-co-glycolic acid) and hydroxyapatite. Biodegradability means that during bone defect repair, new bone tissue can replace materials in the gap, which will degrade at a rate matching that of new bone growth [21, 22]. Here, materials are not only traditional biodegradable polymers and biodegradable ceramics but also callus organoids formed by specific cells, which can be spontaneously bioassembled into large engineered tissues for the repair of tissue damage [23, 24]. With the rapid development of modern science and technology, in the twenty-first century, an increasing number of new biodegradable materials have emerged. However, researchers have not yet developed an optimal strategy for fully matching the degradation rate of the material to the rate of bone regeneration while meeting the different needs of the process of bone tissue regeneration [22].

This review will introduce various kinds of biodegradable materials commonly used in bone defect repair, especially newly emerging materials and related fabrication technologies, and present future research directions, with the aim of providing researchers in the field a reference and some inspiration.

## Bone defects and healing mechanisms

Bone defects refer to bone matrix shortages caused by trauma or surgery, which often lead to non-union, delayed or lack of healing, and local bodily dysfunction [25]. However, there is no clear definition or classification of the severity of bone defects. In general, a “critically sized” bone defect is considered to not spontaneously heal and require manual surgical intervention. At the same time, it has been pointed out that a critical-size bone defect is a defect longer than 1–3 cm with a loss of bone circumference of greater than 50% [26]. However, we must take into account the anatomical location of the defect, the surrounding tissue damage, and the state of the body [7]. Haines et al. [27] showed that defect size and infection degree were key factors affecting the efficacy of treatment. Therefore, we must comprehensively consider various factors that may affect defects to achieve the personalized treatment of clinical bone defects.

Bone formation can be achieved in two ways: intramembranous ossification (IMO) and endochondral ossification (EO), these mechanisms play important roles in natural bone repair after injury and bone development. In short, IMO can increase the number of Osteoblast-related cells in the inner and outer periosteum, make the periosteum thickened and calcified, and then connect the fracture ends; while EO mainly promotes a sterile inflammation reaction between the hematoma at the fractured end and the bone marrow cavity and the surrounding environment, thereby forming granulation tissue, fibrous tissue, and temporary cartilage tissue. In turn, osteoblasts invade and replace chondrocytes, eventually forming bone tissue [2]. The process of bone healing after injury is different from that during natural bone formation (Fig. 1b, c) [2]. After the graft fills the gap and is fixed, the critical-size bone defect is mainly repaired by IMO/EO. According to different ossification strategies, bone grafts made of different materials have been designed to repair bone defects. Some studies have indicated that mineralized biomaterials are effective activators of IMO pathways, including calcium phosphate-based ceramics and other mineralized biomaterials [28, 29]. Unlike mineralized biomaterials, biomaterials (such as naturally derived and synthetic polymers) that enhance cell attachment and subsequent differentiation promote the EO pathway. Although this phenomenon has been reported in many studies, the exact mechanism by which different biomaterials can induce osteogenesis through different pathways is not clear [29, 30]. Because of the need to provide excellent mechanical support and a platform for cell adhesion and nutrient exchange, the porosity and mechanical properties of the scaffold are also critical [31].

In the human body, most bone is grown mainly through the EO pathway, and stem cells are induced to differentiate into functional osteocytes (i.e., osteoblasts) by providing external stimulation to undifferentiated cells, including a mineralized/mineralizable platform, which is similar to the IMO pathway [32]. In recent years, bone regeneration by stimulating EO has received great attention from researchers. In general, biomaterials promote osteogenesis through the EO pathway by locally providing stimulation signals to cells, including undifferentiated or pre-differentiated progenitor cells, various growth factors, and so on [33–36]. A recent study showed that purely biomaterial-based solutions can successfully induce EO to repair critical-size bone defects by mimicking natural extracellular matrix (ECM) [37]. In addition to biomaterials, Nilsson Hall et al. [24] found that callus organisms formed by specific cells that can be spatially bioassembled into multimodular constructs can also repair critical-size bone defects by the EO pathway.

## Biodegradable materials

**Fig. 2:**
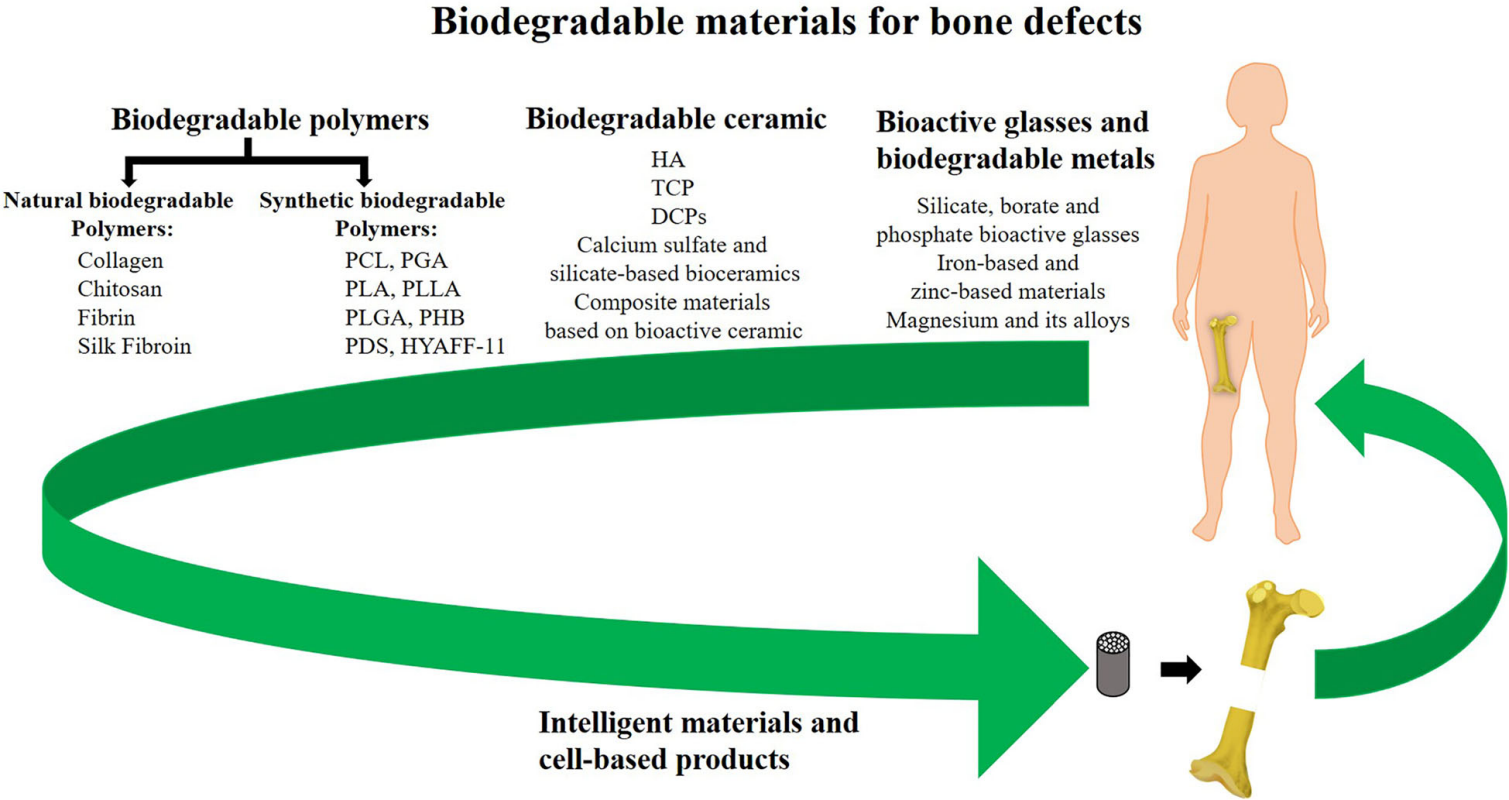
Representation of the main biodegradable materials used for bone defect repair. Biodegradable materials can be divided into three categories: polymer, ceramic and metal materials. In addition, there are newly emerging intelligent materials and cell-based products. Abbreviations can be found in Tables 1 and 2

Biodegradable materials belong to the second generation of biomaterials, which have been closely related to bone defect repair for nearly half a century [16]. Biodegradable materials are widely used in bone tissue engineering because of their biodegradability. As the graft degrades, bone tissue grows into the graft’s interior, and the small biomolecules produced by the degradation can regulate the regenerative microenvironment to adapt to the growth of bone tissue. At the same time, the mechanical properties of the graft gradually decrease, and the biological stress of the body moves from the graft to the new bone tissue, which avoids the stress-shielding effect while stimulating tissue regeneration [38]. Therefore, the degradable biomaterial avoids the injury and related economic burden caused by a second operation. According to the current research status, biodegradable materials are mainly composed of biodegradable polymers, biodegradable ceramics and biodegradable magnesium-based materials (Fig. 2).

### Biodegradable polymers

Polymers generally refer to macromolecules in which repeating monomers are combined by covalent bonds [39]. Among them, biodegradable polymers have been favoured by researchers because of their degradability, which is essential for the repair of bone defects [40]. Depending on their source, polymers can be classified as natural or synthetic. Natural biodegradable polymers, such as chitosan, silk fibroin, fibrinogen, collagen and hyaluronic acid, have been extensively studied as bone defect repair materials due to their biodegradability, bioactivity and biocompatibility. However, they also have some shortcomings, such as source instability, high water solubility, poor mechanical properties, possible denaturation during processing and possible immunogenicity [41]. With their controllable design and synthesis parameters, synthetic polymers can be prepared into biomaterials with excellent mechanical properties [42]. However, when some synthetic polymers are degraded in vivo, their degradation products are acidic and thus change the local pH value, which in turn accelerates the implant degradation rate and induces inflammatory reactions [42]. See Table 1 for abbreviations for biodegradable materials.

**Table 1:** Abbreviations for biodegradable materials

Biodegradable materials	Abbreviations
Chitosan	CS
Poly (ε-caprolactone)	PCL
Poly (glycolic acid)	PGA
Poly (lactic acid)	PLA
Poly (L-lactic acid)	PLLA
Poly (lactic-co-glycolic acid)	PLGA
Poly 3-hydroxybutyrate	PHB
Poly-para-dioxanone	PDS
Benzyl ester of hyaluronic acid	HYAFF-11
Hydroxyapatite	HA
Tricalcium phosphate	TCP
Dicalcium phosphates	DCPs

#### Natural biodegradable polymers

##### Collagen

As the main structural protein of tissues, collagen plays an important role in regulating the extracellular matrix of the cellular microenvironment. Bone is a complex, naturally active tissue that consists of approximately 30% matrix, of which the main constituent is collagen [1].

Collagen is a widely used biomaterial in the biomedical field. Composite membranes based on collagen and apatite crystals have better mechanical properties, so they are receiving increasing attention [43]. At the same time, collagen particles are often added to composite scaffolds to enhance the proliferation of osteoblasts in the bone filler. From a biomimetic perspective, scaffolds made of collagen/bioceramic composite materials can yield better bone repair effects because they are more similar in composition to natural bone [44]. However, the mechanical properties of such scaffolds are often poor, and the collagen needs to be cross-linked. To improve the performance of such scaffolds, other methods have been explored. Recently, Wang et al. [45] prepared novel biomimetic nanosilica-collagen scaffolds by coating acellular porcine cancellous bone porous collagen scaffolds with nanosilica via surface biosilification technology, and these scaffolds led to the successful repair of critical-size cranial bone defects in a rabbit model. The US Food and Drug Administration (FDA) has approved several scaffolds, such as scaffolds made of bovine collagen I, Collagen-graft™ (HA/TCP/bovine collagen), and OssiMend™ (porous bone mineral with collagen) [46]. It is worth noting that Lang et al. found that the use of a biodegradable bovine col-I scaffold alone had a negative effect on bone formation, the possible reason is that in the proteomics analysis, the author found that there may be potential interfering proteins in it. Meanwhile, the author suggested that more complex delivery systems that locally stimulate bone healing should be used in future studies [47].

##### Chitosan

Chitosan (CS) is a natural polymer with a linear structure and is a structural component in the exoskeleton of crustaceans (such as shrimp and crabs). By virtue of its biological activity, biodegradability, antibacterial and biocompatibility, and hydrophilic surface, CS has been reported to enhance cell adhesion, proliferation, osteoblast differentiation and mineralization [48]. Simply put, the cationic properties of CS make it possible to combine with anions that regulate growth factors and cell activity, thereby exerting a physiological role [48]. CS can be formed into 3D scaffolds with different porous structures after advanced preparation processes, such as 3D printing and nanotechnology, and composite materials can be formed with various materials for the repair of bone defects [49]. It is worth noting that no matter what manufacturing process is used, the application of a pure CS bracket in most load-bearing environments is not satisfactory. Therefore, only by blending CS with various natural or synthetic polymers or bioceramics can scaffolds with better biological activity and mechanical properties be obtained. Injectable CS hydrogels can be used to fill irregular bone defects. A recent study showed that Cui et al. [50] designed a kind of interconnected, microporous net of CS cross-linked in situ to form a hydrogel; the added nanosilicate increased the Young’s modulus and slowed down the hydrogel degradation rate.

##### Fibrin

As a natural biopolymer, fibrin is formed in the last step of the coagulation cascade by thrombin acting on fibrinogen [51]. Fibrinogen, thrombin and fibrin precursors can be extracted from human blood as a stable source, which reduces production costs and the risk of unnecessary disease transmission. Considering the critical role of haematoma in the early stage of bone healing, fibrin is a promising choice for incorporation in an ideal scaffold for repairing bone defects. At the same time, fibrin can also promote angiogenesis and osteogenic differentiation, which can in turn accelerate the rate of bone regeneration [52]. However, due to its rapid degradation rate and poor mechanical properties, it is necessary to also use other materials to overcome the limitations of fibrin [53].

Fibrin can be prepared into fibrin hydrogels with injectable properties, but fibrin alone cannot cure bone defects and should be combined with other biomaterials [54]. However, the ability of fibrin glue to promote the bone repair capacity of bioceramics is still controversial, and some scholars have paid attention to the adverse impact of fibrin [55]. Possible reasons include the immune response caused by the use of xenogeneic fibrin and the use of an inappropriate amount of fibrin in the experiment [56]. In addition to modifying scaffolds, fibrin can also be used to transfer cells and growth factors in bone defect repair [57]. A study has shown that fibrin-mesenchymal stromal cell (MSc) composites have an early effect on femoral defects in rats, which supports the attraction of host cells and promotes angiogenesis, thus promoting the process of bone healing [58].

##### Silk fibroin

Silk is a natural protein biopolymer that is mainly produced by silkworms, spiders and some insects to form silk fibre (SF) [59]. Among the different kinds of silk, mulberry silk is the most studied in biomedical research [60]. There are two main protein components in the silk of silkworms: fibroin and sericin. Sericin is degummed during SF purification because it stimulates immune rejection in the host [61]. With its high natural strength, silk has become an important material in the field of bone tissue engineering. According to research, silk-based scaffolds have higher mechanical strength than other naturally biodegradable polymer scaffolds (such as collagen and CS), which makes them popular among researchers in bone tissue engineering [62]. The degradation rate of silk scaffolds is adjustable and usually relatively slow, which helps repair critical-size bone defects [63]. In contrast to the acidic products harmful to tissues produced by the hydrolytic degradation of synthetic polymers, such as poly(lactic acid) (PLA), poly(glycolic acid) (PGA), and poly(lactic-co-glycolide) (PLGA), the proteolytic products of silk-based scaffolds are glycine and alanine, which can be reused as raw materials for new protein synthesis [64].

It has been reported that silk fibroin can promote the expression of early and late cell osteogenic markers, such as runt-related transcription factor 2 (Runx2), osteocalcin (OCN) and osteomodulin mRNA [65]. Silk fibroin can be combined with degradable bioceramics to form large scaffolds of complex shapes with extremely high strength and appropriate porosity to support the growth of cells, thus playing an important role in the repair of bone defects of critical size. Recently, McNamara et al. developed the SF- hydroxyapatite (HA) ceramic scaffolds for load-bearing bone repair with a wide range of mechanical and porosity profiles [66].

#### Synthetic biodegradable polymers

In recent years, the most studied synthetic degradable polymers are aliphatic polyesters, such as poly(ε-caprolactone) (PCL), PLA, PGA and copolymer PLGA [67]. These materials have been proven to be biocompatible and have a controlled degradation rate, and their degradation products in vivo have no toxic effects on tissues. In addition, polymers with improved mechanical properties can be prepared by manually controlling the design and synthesis parameters [42]. Although the acidic degradation products produced by these polymers in the tissue are discharged through the natural metabolic pathway, they may induce an inflammatory foreign body reaction at the local transplantation site, accelerate the degradation rate of the graft and have serious adverse effects on tissue repair, especially in the repair of bone defects in load-bearing areas [68].

##### PCL

PCL is an inexpensive polymer and flexible biologic approved by the FDA. Despite its biodegradability and biocompatibility, after a large number of long-term experiments, researchers found that the degradation rate of PCL was slow and the mechanical properties were poor, so it proved to not be an ideal bone defect repair material [69]. However, a recent study conducted by Rotbaum et al. shows that changing the pore geometry of 3D printed PCL scaffolds can optimize their mechanical properties [70]. Studies have shown that PCL can be used as a material to enhance cell adhesion and proliferation and that applying it to the surface of other composite scaffolds can enhance cell-cell interactions [71]. To improve the availability of PCL in the field of bone defect repair, researchers have tried to combine PCL with bioceramics. A recent study showed that hydroxyapatite-coated PLLA/PCL nanofibre scaffolds could promote the healing of round defects with a diameter of 5 mm in the rat skull within 12 weeks [72].

##### PGA

PGA is a simple aliphatic polyester with a regular linear molecular structure. Glycolic acid is a product of normal human metabolism, and its polymer is PGA. With its excellent tensile modulus and controlled solubility, PGA has been used as the first biodegradable suture in clinical practice for many years [73]. PGA has a high degradation rate, and its degradation product, glycolic acid, can be excreted through urine [46]. Compared with other degradable polymers (such as PCL and PLA), PGA has higher mechanical strength [74]. Specifically, the young’s modulus of PGA, PCL and PLA are 5-7GPA, 0.4–0.6GPA and 2.7GPA [46]. However, due to its excessively rapid degradation rate in vivo, a PGA scaffold alone is not suitable for repairing bone defects [75]. Therefore, many researchers have prepared PGA composite scaffolds together with other materials and evaluated their application in bone defect repair. Toosi et al. evaluated the role of a collagen/PGA scaffold in the regeneration of rabbit skull defects and found significant fibrous connective tissue formation after 12 weeks of treatment [76].

##### PLA

PLA is a polymer consisting of lactic acid and was first discovered and named by a Swedish chemist named Scheele in 1780 [46]. Meanwhile, PLA is a biodegradable polymer made from starch sourced from renewable plant resources (such as sugar cane and corn) [46]. At present, L-PLA and DL-PLA (mixture of L-and D-lactic acid) are the most widely used PLA in clinical [77]. Because of its high mechanical strength, porous structure, and sufficient porosity, L-PLA is often used to prepare scaffolds for bone tissue engineering applications [78]. One study found that PLA-PCL tissue-engineered scaffolds loaded with BMP-2 had good bone repair effects [79]. At the same time, PLA can also be combined with biodegradable ceramics to prepare scaffolds. Zhang et al. found that when the mass ratio of PLA/HA was 8:2, the overall performance of the prepared porous scaffold was the best [80]. Recently, the biomimetic mineralized strontium-doped hydroxyapatite on porous poly(l-lactic acid) (Sr-HA/PLLA) porous scaffold prepared by Ge et al. can reduce the degradation of the acidic environment, improve the hydrophobicity of the surface of the material, increase the protein adsorption capacity of the material and increase the osteoinducibility of the material [81].

##### PLGA

PLGA is formed by the ring-opening copolymerization of PLA and PGA, and its degradation rate can be regulated by changing the percentage of these two polymers [82]. PLGA is a widely used biodegradable polymer that has the advantages of safety, biocompatibility, non-cytotoxicity, ideal mechanical properties and controllable degradation [46]. Therefore, PLGA is more popular with researchers than PGA and PLA and can be used to prepare sutures and cell and drug delivery systems [83]. However, despite the above advantages, the utility of PLGA is limited in bone repair because of poor osteoconductivity and hydrophobicity [84, 85]. Fortunately, these shortcomings can be compensated for by other materials. Lai et al. prepared a porous PLGA/TCP/Mg (PTM) scaffold using low-temperature rapid prototyping (LT-RP) technology; this scaffold promoted both osteogenesis and angiogenesis and significantly promoted the formation of new bone in bone defects in rabbits [86].

##### PHB

Poly(3-hydroxybutyrate) (PHB) is a kind of polyhydroxyalkanoate (PHA) that was first isolated by the French microbiologist Maurice Lemoigne in 1925 [87]. As a highly biocompatible polymer, PHB can be degraded in tissue through enzymatic and hydrolytic degradation. Unlike other common biodegradable polymers, such as PGA, PLGA or PLA, when PHB degrades, the local pH remains in a stable range [88]. Compared with materials with stronger mechanical properties, PHB has lower strength and lower rigidity, which is like a double-edged sword [89]. Because PHB allows slight movement after implantation, this may be beneficial to bone healing [90]. Meischel et al. implanted PHB composites into the femur of SD rats and found no significant degradation after 36 weeks in vivo, suggesting that the tensile strength, strain properties and elastic modulus of PHB composites are similar to those of natural bone, which may thus be a promising material for bone defect repair [91]. Among PHA, besides PHB, copolymers of 3-hydroxybutyrate and 3-hydroxyvalerate (PHBV) is another widely studied polymer [89]. With the help of electrospinning technology, Zhang et al. modify the surface of PHBV and fabricated PHBV-CS-hydroxyapatite biocomposite nanofiber scaffolds, on which the proliferation rate of osteoblasts was 34.10% higher and the mineral deposition was higher (25.79%) than that of PHBV scaffolds on the 20th day [92].

##### PDS

Poly(para-dioxanone) (PDS) is a biodegradable polyester obtained by the ring-opening polymerization of a hydroquinone monomer. With excellent biodegradability and biocompatibility, PDS is very popular in tissue engineering and fracture repair [93]. When used for internal fracture fixation, PDS can be completely absorbed and degraded by bone tissue [94]. Unfortunately, there have been no studies on the use of PDS for bone defect repair.

##### HYAFF-11

HYAFF-11 is a benzyl ester derivative of the polysaccharide hyaluronic acid, commonly found in the extracellular matrix [95]. With good biocompatibility and a degradation rate that can be controlled by the esterification degree, HYAFF-11 is a very promising material for tissue repair [96]. At present, HYAFF-11 has been used in skin repair, nerve regeneration, bone substitutes and other fields [97, 98]. A recent study revealed that the HYAFF-11 membrane prepared by Mermerkaya et al. plays an important role in repairing 10-mm rabbit tibial defects and enhances osteogenic activity during early bone healing [99].

### Biodegradable ceramics

Ceramic is made of natural clay and various minerals as the main raw materials combined through crushing, mixing, moulding and calcining [100]. In the past few decades, bioceramics have been widely used in the repair and replacement of damaged tissues due to their advantages of biocompatibility, mechanical compatibility and precise chemical composition [101]. Among them, biodegradable ceramics used in bone tissue engineering are more favoured by researchers. Specifically, they are mainly used for filling the gaps of bone defects and repairing fractures [102]. At present, the most frequently used biodegradable ceramics mainly include hydroxyapatite (HA), tricalcium phosphate and dicalcium phosphates. Biodegradable ceramics have the advantages of biocompatibility, corrosion resistance and biological activity. The greatest advantage is that they are gradually degraded by solution-driven and cell-mediated processes after implantation in the body and finally replaced by new lamellar bone tissue. Of course, biodegradable materials also have some shortcomings, such as poor fracture toughness, brittleness, and extremely high rigidity, and their strength is significantly lower than that of non-absorbable ceramic materials [103].

#### Hydroxyapatite

Hydroxyapatite (HA), known as Ca_10_(PO_4_)_6_(OH)_2,_ is a widely used bioactive and biodegradable calcium phosphate that accounts for almost 65% of the total bone mass and constitutes most of the inorganic components of bone tissue [46]. Calcium orthophosphate ceramics have a calcium/phosphorus molar ratio between 0.2 and 2.0; HA has a calcium/phosphorus ratio of 1.67 [104]. Synthetic HA is a highly crystalline form of calcium phosphate, which is usually prepared by a high-temperature reaction. Since natural HA is the most inorganic component of bone tissue, synthetic HA and natural HA have a high chemical similarity, so synthetic HA shows good osteoconductivity. However, they are slightly different in physical microstructure, crystal size and porosity [105]. After implantation into the gap of bone defects, HA can directly combine with new bone tissue, promote graft vascularization and stem cell proliferation and guide bone regeneration [104]. The biodegradation rate of HA is relatively slow, which may have a negative impact on bone defect repair. Specifically, the HA surface is often covered by bone after implantation without inter-position of connective tissue, which will hinder the degradation and absorption of the material. Brandt et al. implanted nano-crystalline HA into the distal femur of the rabbit, and then observed no significant absorption at the edge of most grafts [106]. In view of the above shortcomings, various modifications of HA have been explored by researchers. HA-based bone grafts doped with manganese and zinc have shown faster degradation rates [46, 107]; adding Sr^2+^ or Mg^2+^ can improve the mechanical and biological properties of HA-based bone substitutes [108], and the possible reason is that the change of physical and chemical properties of HA crystallinity, microstructure and solubility caused by the introduction of cations [109–111]. A recent systematic review has shown that HA bone grafts can enhance the healing of critical- and non-critical-size bone defects [112].

#### Tricalcium phosphate

Tricalcium phosphate (TCP), Ca_3_(PO_4_)_2_, is a common absorbable bioactive ceramic material with a calcium/phosphorus ratio of 1.5. TCP has three crystalline forms, α-TCP, β-TCP and α′-TCP [113]. Due to the similarity in terms of crystallinity and chemical composition with bone minerals, β-TCP has excellent biocompatibility and biodegradability and is favoured by researchers. The young’s modulus of TCP and HA are 60-75GPA and 80–110 GPA, respectively [114, 115]. Although the mechanical strength of β-TCP is slightly lower than that of HA, its biodegradation rate is significantly faster than that of HA, which is beneficial to the growth of new bone around implanted TCP-based scaffolds [116]. It is pointed out that the degradation rate and bioactivity of biphasic calcium phosphate (BCP) bioceramics mainly composed of HA and TCP depend on the ratio of HA/TCP, which is inversely proportional to the ratio of HA/TCP [117]. A study of the filling of a bone defect in the goat radial shaft with porous β-TCP (prepared by an aqueous solution combustion technique) showed extensive bone formation throughout the defect 3 months after the operation [118]. According to research, S1P can up-regulate the expression of OPN, OCN and RUNX2 genes related to osteogenesis, and significantly increase alkaline phosphatase activity; collagen is an important component of natural bone; iron ions can affect collagen maturation and vitamin D metabolism [119, 120]. Therefore, TCP can be combined with other materials to enhance its biomechanical properties and osteogenic ability, such as collagen [121], sphingosine 1-phosphate (S1P) [119] and metal ions [122].

#### Dicalcium phosphate

Dicalcium phosphate (DCP) is a kind of acid calcium phosphate with a basic calcium source and acidic phosphorus source [46]. As the main component of calcium phosphate cement (CPC), DCP has two forms, namely, monetite [CaHPO_4_, dicalcium phosphate anhydrous (DCPA)] and brushite [CaHPO_4_·2H_2_O, dicalcium phosphate dihydrate (DCPD)] [123]. Among calcium phosphate ceramics implanted in the body, DCPD has relatively high solubility [120]. At the same time, DCPD bone cement (brushite bone cement) has been approved for clinical application in Europe for many years [124]. However, in recent years, studies have reported that brushite bone cement rapidly degrades after implantation, and its degradation products are converted into insoluble forms of apatite, such as HA, in the body, which affects its role in repairing bone defects [125]. Recently, Shariff et al. reported that coating an appropriate amount of acidic calcium phosphate solution (DCPD) on the surface of β-TCP to prepare a new material can improve the osteoconductivity of β-TCP, and a large amount of new bone formation was observed 4 weeks after its implantation in rats [126].

According to research, compared with brushite bone cement, monetite has a greater potential for resorption and bone formation. One possible explanation is that compared to brushite, monetite is less soluble and lacks the tendency to convert to HA [127]. In addition, a recent study based on critical-size bone defects in the rat radius also reported the excellent biocompatibility, biodegradability and biomechanical properties of DCPA or monetite ceramic materials [128].

#### Calcium sulfate and silicate-based bioceramics

Calcium sulfate is a mineral, which exists in the form of gypsum ore in nature and is composed of calcium sulfate dihydrate (CaSO_4_·2H_2_O) [129]. In the calcination process heated to 110 °C, calcium sulfate loses water to form α and β two forms of calcium sulfate hemihydrate (known as Plaster of Paris) [130]. The research on repairing bone defects with calcium sulfate was first reported by Dreesman in 1959 [131]. An experiment using calcium sulfate to repair canine alveolar bone defects showed that calcium sulfate can significantly improve alveolar bone and cementum regeneration [132]. In addition, calcium sulfate can upregulate bone formation-related genes in vitro and improve osseointegration in vivo [133]. Although calcium sulfate has the above advantages, long-term studies have found that its degradation rate is too fast, which cannot match the regeneration rate of new bone tissue. At the same time, it may cause adverse reactions such as inflammation and surface instability [115, 134]. Meanwhile, due to the low mechanical strength, calcium sulfate cannot provide sufficient long-term mechanical support for the defect [135]. Therefore, scholars have carried out various treatments on calcium sulfate to make it better used in tissue engineering. Cui et al. [136] coated chitosan with calcium sulfate and then compounded with BMP-2 to form composite particles, which had better compressive strength and osteoinductivity (provided by BMP-2). At the same time, the results of in vivo repair of rabbit radial defects showed that the absorption time was longer than that of uncoated calcium sulfate particles. In addition, Hao et al. [137] mixed tricalcium silicate into calcium sulfate to prepare composite bone cement, which was used to repair a femoral condyle defect in rabbits. In vivo experiments showed that calcium phosphate bone cement was completely degraded after 8 weeks of implantation, and composite bone cement was only 50% degraded after 12 weeks of implantation. Calcium sulfate can also be used to prepare new injectable biomaterials. Chen et al. [138] introduced calcium sulfate hemihydrate into mineralized collagen to prepare an injectable and controllable bone repair material, of which the degradation rate matched the growth rate of new bone tissue in the mandible transplantation site of rabbit.

The content of silicon in the earth’s crust is the most element other than oxygen, which mainly exists in the form of complex silicate or silica. At the same time, silicon is one of the essential trace elements of the human body, accounting for about 0.026% of body weight [139]. Silicon plays an important role in connective tissues such as articular cartilage and bone [140]. In addition, according to the researches, silicon can promote the proliferation and differentiation of rat bone marrow stromal cells, and promote the collagen synthesis process of osteoblasts [141, 142]. Due to the important role of silicon in bone growth and mineralization, silicate bioceramics are widely used in bone tissue engineering [143]. However, it is pointed out that CaSiO_3_ ceramics have the disadvantage of high dissolution rate, resulting in high pH in the surrounding environment, which is not conducive to cell growth and limits its application in the field of bone tissue engineering [144–146]. Therefore, the author added zinc to CaSiO_3_ to prepare a new crystal phase (hardystonite), which has the best chemical stability and cell biological activity in zinc-containing calcium-silicon ceramics [147]. A series of studies have shown that many silicate bioceramics can stimulate the osteogenic differentiation of bone marrow stromal cells (BMSCs) and adipose stem cells (ADSCs), such as akermanite (Ca_2_MgSi_2_O_7_) [148], baghdadite (Ca_3_ZrSi_2_O_9_) [149], hardystonite (Ca_2_ZnSi_2_O_7_) [147], diopside (CaMgSi_2_O_6_) [150]. Specifically, Gu et al. [151] confirmed through research that the akermanite dissolved ion products (Ca, Mg and Si) promotes the osteogenic differentiation of human fat stem cells by activating the ERK pathway. Luo et al. [152] used the microsphere-shaped diopside (CaMgSi_2_O_6_) and baghdadite (Ca_3_ZrSi_2_O_9_) to fill the supracondylar bone defect in rats. In vivo experiments showed that the baghdadite microspheres had a higher content of new bone and the expression of osteopontin.

#### Composite materials based on bioactive ceramics

Composite materials based on bioactive ceramics mainly refer to materials with the complementary advantages of both biodegradable polymers and biodegradable ceramics. In general, these composites possess excellent biocompatibility, osteoconductivity, mechanical strength, and osteogenic characteristics. At the same time, with the help of new fabrication techniques that have emerged in recent years, these composite materials have become the most promising materials in the field of bone defect repair.

A recent study has shown that an innovative collagen/HA hybrid scaffold can induce the osteogenic differentiation of human BMSCs, induce the upregulation of osteogenic gene expression, and increase collagen deposition [153]. Similarly, satisfactory results have been observed in other studies of collagen/HA composite material [154]. Another recent study has shown that PCL/silicon-substituted hydroxyapatite (Si-HA) membranes can induce cell growth and differentiation and improve osteoblast attachment and proliferation; thus, this material is expected to play an important role in bone defect repair [155]. In addition, new materials prepared by combining multiple materials with improved biological properties are also emerging. Recently, in order to repair the bone defect caused by steroid associated osteonecrosis (SAON), Lai et al. [86] prepared a new porous PLGA/TCP /Mg (PTM) scaffold with magnesium powder, PLGA and β-TCP. The in vivo experimental results show that the PTM scaffold has the dual effects of osteogenesis and angiogenesis, and at the same time has a synergistic effect in promoting the formation of new bone and improving the quality of new bone in SAON.

### Bioactive glasses

In the early 1970s, Professor Hench developed a silicate-based 45S5 glass based on the system of SiO_2_ (45%)-Na_2_O (24.5%)-CaO (24.5%)-P_2_O_5_ (6%) [156]. Since then, bioactive glass (BAG) began to enter people’s field of vision and played an important role in the repair of bone defects [157]. When 45S5 was implanted into the body and contacted with body fluid, HA layer similar to the host bone could be formed on the surface of the glass, and then formed a strong chemical bond with the host bone [158]. However, silicate BAG has a strong tendency to crystallize, the degradation rate is slow and cannot match the rate of new bone formation, and it cannot be completely converted into HA. Therefore, the application of silicate BAG in bone regeneration and repair always has certain limitations [159]. In order to overcome the shortcomings of silicate BAG, borate BAG was developed in 1990 [160]. Compared to silicate BAG, borate BAG is more chemically active. The B_2_O_3_ content in the components can be artificially adjusted to achieve a rate of material degradation that matches the rate of new bone formation; as it can be almost completely converted to HA, the borate BAG’s osteogenic ability is also more excellent [161]. With the development of the research, it is found that the rapid dissolution of (BO_3_)^3−^ from borate BAG has a certain toxic effect on cells [160]. In addition, it is found that phosphate BAG is another kind of BAG with high activity and faster degradation rate. It can play the role of local anti-infection, osteogenesis and angiogenesis by mixing various functional elements (such as strontium, silver and zinc) [162, 163].

There are many forms of BAG used in bone tissue engineering, such as particles [164], coating [165], bone cement [166] and scaffolds [25]. At the same time, BAG can also be used to load drugs [167] and biological factors [168]. Excitingly, there are already several particulate BAG products in clinical use, such as PerioGlas®, NovaBone® and BonAlive®. According to relevant research, PerioGlas® is the first particulate BAG product to be used clinically, which is mainly used to strengthen periodontal tissues and repair jaw defects [169]. When treating patients with idiopathic scoliosis, NovaBone® can achieve the same effect as autogenous bone transplantation in spinal fusion and orthodontics [170]. During the 11-year follow-up after treatment of tibial fractures, the researchers found that BonAlive® had a similar bone regeneration effect as autogenous bone transplantation, with some glass particles remaining. In another treatment for bone defects (1–30 cm^3^) due to benign tumour resection, some glass particles of BonAlive® remained after 14 years of follow-up [171]. However, particulate BAG has some disadvantages, such as low mechanical strength, which can only be used to repair bone defects in non-load-bearing parts; slow degradation in vivo, the degradation rate does not match the rate of new bone formation; it is unable to add other components, so it cannot play other roles in bone repair.

A recent study showed that Ravanbakhsha et al. [164] prepared mesopore bioactive glass (MBG) sub-particles by the sol-gel method, which has good bone-forming ability and is more likely to form an HA layer after contact with body fluids. At the same time, its highly ordered pore structure makes it easy to load drugs, and has become a good candidate for drug delivery. The BAG coating of the prosthesis can form a chemical bond with the host bone interface at the early stage of implantation. At the same time, the BAG coating can also protect the prosthesis matrix from corrosion and prevent the prosthesis from releasing toxic metal ions [165, 171]. In recent years, the new bone cement prepared by BAG has attracted great interest from researchers. Zhang et al. [166] prepared a novel injectable bone cement (Sr-BBG) composed of strontium-doped borate BAG particles and chitosan, which shows the better mechanical properties and bone forming ability due to incorporation of strontium. For the degradation of BAG scaffolds, some scholars have also made in-depth research. Recently, Niu et al. [168] made an in-depth evaluation of the resorption/osteogenesis properties of the rhBMP-2-loaded trimodal macro/micro/nano-porous bioactive glass scaffold (TMS-rhBMP-2). The in vivo results of rabbit radius large segmental defect model show that the TMS-rhBMP-2 has similar biodegradation rate (2.43, 1.81, 0.54 and 0.32%/day) and bone formation rate (2.85, 2.14, 0.78 and 0.46%/day) at 0–1, 1–4, 4–8 and 8–12 week. At the same time, a long-term MRI result showed that the bioactive glass substrate in TMS-rhBMP-2 was mostly degraded by the 8th week and completely absorbed by the 12th week.

### Biodegradable metal materials

Metal implants have a long history of application in orthopaedic surgery, especially in the field of bone repair, with common implant materials including stainless steels, titanium and cobalt-chromium-based alloys [172]. However, these materials have many shortcomings, such as non-biodegradability and stress-shielding effects, which limit their application in bone defect repair. In recent years, biodegradable metals have attracted extensive attention from researchers due to their excellent biocompatibility and degradability [173, 174]. Specifically, the most widely studied biodegradable metals include magnesium, iron, zinc and their alloys. At the same time, these three metals are essential elements for maintaining the normal function of the human body, which has been confirmed by many studies to have good biocompatibility to human cells and tissues [175–177].

#### Biodegradable magnesium-based materials

With good biocompatibility, suitable mechanical strength and biodegradability, magnesium and its alloys are widely favoured by researchers in the field of bone regeneration [178]. Among the cations in the human body, magnesium is ranked fourth and is mainly stored in bone tissues, participating in many metabolic processes in the body [46]. The biomechanical properties of magnesium are suitable for bone tissue. The density of magnesium-based metals (1.7–1.9 g/cm^3^) is very similar to that of human cortical bone (1.75 g/cm^3^) [105]. The elastic modulus of magnesium-based metals is ~ 45 Gpa, which is relatively close to that of natural bone (3–20 Gpa), while the density of titanium alloy and stainless steel is 4.47 and 7.8 g/cm^3^, respectively, and the elastic modulus is 110 and 200 GPa [179]. Therefore, compared with commonly used titanium alloys and stainless steels, magnesium-based metals only have a negligible stress-shielding effect.

The greatest advantage of magnesium is its biodegradability. Under the action of Cl^−^ in the tissue microenvironment, magnesium is degraded; the degradation product, Mg^2+^, can be excreted through urine [180]. At the same time, magnesium has excellent biocompatibility. To date, there have been no reports on the critical toxicity limit or side effects of Mg^2+^ [181]. However, to be a clinically qualified bone graft material, the degradation rate must match the regeneration rate of bone tissue. The healing of bone tissue usually includes three stages: the early inflammatory stage (3–7 days), the repair stage (3–4 months), and finally the continuous remodelling stage (months to years) [182]. Therefore, a qualified bone implant must maintain sufficient mechanical strength for at least 12 weeks. However, in most current studies, magnesium and its alloys cannot maintain sufficient mechanical strength because of rapid degradation after implantation. Specifically, the degradation rate of magnesium is affected by complex environmental factors in vivo, such as Cl^−^, Ca^2+^, PO_4_^−^, proteins and other organic molecules in blood [183]. In addition, the rapid degradation of magnesium will release a large amount of hydrogen [184], which can accumulate to form air pockets near the implant, potentially leading to tissue and tissue layer separation, delayed bone defect repair and tissue necrosis [185]. Although the body can maintain the pH of body fluids and blood at a steady state, the rapidly degrading Mg raises the pH around the implant site, which can have a serious impact on bone regeneration [186]. If the local pH value of the graft in the body exceeds 7.8, it may cause an alkaline poisoning effect [172]. There have been different attempts to control the degradation rate of magnesium, including purification, alloying, and surface modification.

According to recent research, the new degradable magnesium alloy ZEK100 and tricalcium phosphate-coated magnesium alloy AZ31 both have good biocompatibility and biodegradability [187]. Compared with purification and alloying, the surface modification operation is simpler and more convenient, and at the same time, it can reduce the degradation rate and improve the surface biocompatibility of Mg and eliminate the addition of potentially toxic alloying elements [188, 189]. According to current research, a variety of surface modification strategies can achieve satisfactory results, such as plasma electrolytic oxidation (PEO), HA coating, sol-gel coating, organic coating, electrodeposition, chemical deposition and biomimetic treatment [190, 191]. In order to reduce the rapid degradation of magnesium in the physiological environment, Wu et al. [192] recently modified the surface of pure magnesium by ion electrolytic oxidation and hydrothermal treatment technology and formed a dense protective layer. The results of in vivo repair of rat skull defects showed that the biodegradation of the surface modified magnesium grafts slowed down significantly. In addition, Li et al. [193] adopted the sandwiched biocompatible coating strategy to apply stearic acid coating on magnesium alloy, which has better corrosion resistance and biocompatibility. Of course, before the above materials can be used in the future, strict and standardized in vivo experiments and long-term implant studies are needed to determine whether the biodegradability, biocompatibility and mechanical strength of these new magnesium materials meet the clinical standards.

#### Biodegradable iron-based and zinc-based materials

Iron is an essential trace element in the human body. The total amount of iron in the human body is about 4–5 g, which is an important part of haemoglobin [194]. Some in vivo and in vitro experiments have shown that magnesium has good biocompatibility [195, 196]. At the same time, iron metal, with excellent mechanical properties close to 316 L stainless steels, plays an important role in the field of tissue engineering [197]. Compared with pure magnesium, pure iron has stronger mechanical properties, making it an implant that requires high structural strength such as bone defect repair and vascular stents [198, 199]. In addition, iron is relatively easy to obtain and inexpensive, and it does not release hydrogen during the biodegradation process after implantation [200]. However, research shows that the main disadvantage of pure iron and iron-based materials in application is the slow degradation rate [195]. A study of descending aorta implanting a corrodible stent produced from pure iron in pigs showed that although there were signs of degradation after 1 year, most stents were still intact [201]. Therefore, improving iron degradation rate is an urgent task to promote the use of iron-based stents in clinical practice. To this end, researchers have made a variety of attempts, such as surface modification [202], alloying [203], and adding a second phase [204]. Additively manufactured (AM) porous biomaterials can increase the surface area of the material [205]. Generally speaking, a larger surface area usually leads to a higher biodegradation rate. Therefore, for iron and its alloys, increasing the surface area may be a promising way to accelerate its biodegradation rate [206]. Recently, Li et al. used direct metal printing (DMP) technology to prepare AM porous iron scaffolds [199]. Electrochemical tests have shown that the biodegradation rate of AM porous iron is 12 times that of cold-rolled iron. At the same time, after 28 days of degradation, the mechanical properties of AM porous iron (Elastic modulus = 1600–1800 MPa) are still similar to those of trabecular bone.

Similar to iron, zinc is also an important trace element required by the human body and plays an important role in many physiological activities (such as growth, immunity, and wound healing) [207, 208]. It is reported that about 85% of zinc is present in muscles and bones, so zinc is essential for bone development and growth [209]. In order to maintain the normal zinc demand of the body, the recommended daily intake of zinc is 15–40 mg [210]. A series of in vitro studies have shown that zinc ions can promote stem cell osteogenesis and increase mineral deposits, as well as promote osteoblast adhesion, proliferation and differentiation [211–213]. A study of vascular stent transplantation in rat abdominal aorta in 6 months showed that the biodegradation rate of pure zinc stent was faster than that of Fe and Mg alloy [214]. However, due to the soft texture and low mechanical strength of pure zinc (tensile strength was below 20 MPa, elongation was only 0.2% and vickers hardness was 37), there are few reports of pure zinc scaffolds for bone tissue engineering [207]. Compared with pure zinc, Zn alloy prepared by adding other metal elements (such as Mg, Ca and Sr) shows significant improvement in mechanical properties and biocompatibility [215]. In addition, in vivo experiments show that Zn-Sr alloy has a good role in promoting new bone formation. Recently, Tiffany et al. [216] added zinc to the mineralized collagen suspension, and then lyophilized to form a porous zinc-containing mineralized collagen bone scaffold. In addition, Mg-Zn-Ca-alloy scaffold prepared by Zhang et al. [217] showed good corrosion resistance and osteogenic performance, and showed satisfactory bone repair effects in the rabbit ulnar defect model.

## Fabrication technologies for improved biodegradable materials

With numerous great properties, including osteoinductivity, osteointegration and osteoconductivity, autogenous bone transplantation is still the gold standard for bone defect repair and regeneration at this stage [9]. At the same time, autogenous bone also has all the basic elements to promote bone regeneration, such as an appropriate porosity, excellent surface topography, non-immunogenic autologous stem cells and various necessary growth factors [218]. From the viewpoint of tissue engineering, we can simply understand bone tissue as a 3D nanoscaffold composed of nano-HA and self-assembled collagen fibres (Fig. 1a) [1]. Therefore, based on the strategy of biomimetics, researchers are committed to making excellent artificial bone grafts that can simulate autogenous bone to the greatest extent at the macro, micro and nano scale with the help of all available new technologies. According to current research, 3D printing technology can be used to manufacture complex, unique 3D structured scaffold with a suitable porosity for bone defect repair [21]. In addition, interfacial reinforcement, especially nanotechnology, can provide the scaffold with an appropriate surface topography, surface microroughness, surface hydrophilicity and surface charge [219]. Specifically, the porosity and pore size of the biomaterial scaffold can play an important role in the repair of bone defects by affecting the mechanical stability of the scaffold, the migration and proliferation of osteoblasts and mesenchymal cells, and vascularization [220]. The surface nanotopography mainly influences cell recruitment, cellular adhesion, osteogenic differentiation, mineralization, osseointegration, and osteoimmunomodulation [221, 222]. See Table 2 for abbreviations for fabrication technologies.

**Table 2:** Abbreviations for fabrication technologies

Fabrication technologies	Abbreviations
Stereolithography	SLA
Fused deposition modeling	FDM
Selective laser sintering	SLS
Inkjet-based bioprinting	IBB
Extrusion-based bioprinting	EBB
Laser-assisted bioprinting	LAB
Interface phase introduction	IPi
In situ growth	ISG
Surface modification	SM

### 3D and 4D printing

Tissue engineering includes three elements: scaffold, seed cell and growth factor. Scaffolds, as the main component of the three elements of tissue engineering, play an important role in defect repair. Scaffolds are a combination of degradable biomaterials that serves as a bridge for new bone tissue to fill in the gap of bone defects and a platform for growth factors and cells to function [223]. To become a qualified bone repair scaffold, in addition to biodegradability, biocompatibility, osteoconductivity and mechanical strength, interconnected porous structures are particularly important. Studies have shown that scaffolds with a porosity greater than 90% and a pore diameter from 300 to 500 μm are conducive to cell infiltration, vascularization and nutrient exchange [21]. However, some recent studies have raised different points of view. A recent study on scaffolds with different porosity found that the cell proliferation rates of two types of scaffolds with porosity of 30 and 50% were satisfactory and equal. Therefore, they suggest that the recommended porosity of scaffolds for bone defect repair may not need to be maintained at about 90% [224]. At the same time, the optimal pore size is different for different types of materials used to fabricate 3D scaffolds [225]. For example, for PLA, if we want to obtain the optimal vascularization of regenerated tissue, the most suitable pore size is 300 μm [21]; for PLA and collagen composite, if we want to obtain the optimal vascularization and mechanical properties of regenerated tissue, the most appropriate pore size is 600 μm; at the same time, the authors note that in vitro experiments, the scaffold with the characteristics of 600 μm pore can promote cell proliferation and adhesion to a greater extent than that with 900 μm pore [225].

In the past few decades, many techniques have been used to prepare scaffolds for tissue repairs, such as freeze-drying, gas foaming, electrospinning, solvent casting and phase separation [226, 227]. However, these scaffold preparation technologies cannot produce satisfactory scaffolds with a suitable porous structure, porosity and pore size. In detail, these techniques cannot accurately control various parameters of the scaffold according to the researcher’s purpose, and there is a certain degree of randomness [228]. Fortunately, additive manufacturing, also known as 3D printing, has become an excellent manufacturing method in recent years, especially in the field of scaffold preparation, which opened new prospects for the repair of critical-size bone defects [229].

For a long period of time, conventional 3D printing technologies have contributed to the preparation of bone repair scaffolds, such as fused deposition modelling (FDM), stereolithography (SLA) and selective laser sintering (SLS) [230, 231]. With high manufacturing accuracy, SLA is often used to make bone repair scaffolds [231]. The photocrosslinkable poly(trimethylene carbonate) (PTMC)-HA nanoparticle scaffolds prepared by Guillaume et al. [232] by SLA have a rich microscale layer and can promote osteogenesis in vitro and in vivo. Because the working principle of FDM is to extrude the material from a small nozzle after melting at high temperature and then allow it to harden to form a solid structure, FDM can be used to prepare complex 3D scaffolds with a controllable pore size and interconnected pores [233]. According to current research, a variety of biodegradable materials can be prepared as bone repair scaffolds with excellent mechanical strength by FDM, such as PCL, PLA and PLGA [234]. In SLS, an infrared laser is used to selectively sinter powder materials and form a solid structure after excess powder is removed. With SLS technology, biodegradable materials, such as PCL, collagen and β-TCP, can be prepared as bone repair scaffolds for non-load-bearing areas, which have similar mechanical properties to trabecular bone (compressive strength: 80–150 MPa) [230, 235, 236]. However, these conventional 3D printing technologies have some disadvantages. Firstly, only a few materials are suitable for SLA, while others are limited by viscosity and stability [231]. Secondly, FDM can only be used to prepare scaffolds with regular shapes, cannot be applied with temperature-sensitive materials, and is limited by a low spatial resolution and high operating temperature [234]. Finally, it is impossible to add bioactive materials, such as cells and growth factors, during the preparation of SLS scaffolds with the limitation of the extremely high operating temperature [237].

With the rapid development of science and technology in recent years, emerging 3D bioprinting technology has shown great advantages in the preparation of porous bioactive scaffolds with a controlled cell distribution, especially for bone tissue engineering. Based on current research, common 3D bioprinting methods include inkjet-based bioprinting (IBB), extrusion-based bioprinting (EBB) and laser-assisted bioprinting (LAB) [238, 239]. IBB is performed using the most traditional and widely used desktop inkjet printers; the main working principle is to use pulse pressure generated by piezoelectric or heat to drive the ejection of biological ink from the nozzle for biological printing [240]. IBB can be used for the preparation of scaffolds with various materials, such as ceramics, degradable metals and polymers. In EBB, bioink is extruded through a micronozzle based on a continuous extrusion process. Meanwhile, EBB has a wider selection of bioink, and the preparation process does not involve heating, so it can be used to prepare composite scaffolds with cells and bioactive materials [241]. A series of degradable biomaterials can be used as bioinks for EBB, such as Methacrylated gelatin (GelMA)/gelatine bioinks and rhBMP-loaded calcium phosphate nanoparticle/PLLA bioinks, and the 3D scaffolds prepared with them have a good porous structure and osteoinductivity. The authors pointed out that compared with high concentration of GelMA, low concentration of GelMA does not induce the abundant covalent bonds. Therefore, the scaffold prepared from low-concentration GelMA has a higher porosity, and the bone marrow stromal stem cells cultured on the surface thereof show a higher cell diffusion rate and cell activity [242]. LAB technology is based on the principle of laser-induced forward transfer (LIFT). LIFT is a direct write technology, the basic principle of which is that the laser energy heats the absorption layer, which is then transferred to the bioink film on top of the absorption layer, generating a jet and then transferring the material to the receiving substrate [234]. Compared with IBB and EBB, LAB does not have the problems of nozzle clogging and cell damage or death caused by potential shear stress [243]. At the same time, LAB has a higher resolution. A recent study reported that LAB can be used to print collagen/nano-HA directly into critical-size defects in mouse skulls [244].

4D printing refers to the preparation of 3D objects with physical properties (including shape, density, elasticity, conductivity and electromagnetic properties) that can self-transform under predetermined stimuli (such as heat, pressure, electricity, and light) by using “programmable materials” and 3D printing technology [245]. “Programmable materials” are materials that can programmatically change in terms of shape, density, elasticity, conductivity and electromagnetic properties. A recent study reported a shape-memory, porous (SMP) scaffold loaded with bone morphogenetic protein-2 (BMP-2) prepared by chemically cross-linked PCL and HA nanoparticles. Excitedly, the volume of the SMP scaffold was smaller under thermal compression, and its original shape could be restored after implantation and exposure to body temperature. The results showed that the SMP scaffold had good cell compatibility and shape-memory recovery in vivo and in vitro and could promote the formation of new bone in rabbit mandibular defects [246]. In other recent research, 4D-printed shape-memory functional tracheal-bronchial stents have also been implanted in infants with tracheobronchial softening [247]. 4D shape-memory scaffolds have also been used for the study of cardiovascular diseases in rats and pigs [248]. In 1957, Fukada and Yasuda [249] discovered the piezoelectric effects in bone tissue. Some studies have shown that bioelectric signals and endogenous electric fields can regulate cell behaviour and promote bone repair [250]. Piezoelectric materials can transmit electrical signals, which in turn can enhance the physiological electric environment to stimulate tissue repair. At the same time, piezoelectric materials can be driven by physiological electrical changes, thereby generating mechanical signals [251]. An early study showed that piezoelectric materials-PLLA can increase callus formation around piezoelectric implants [252]. In addition, a recent study has shown that the cell adhesion can be improved by increasing the surface energy and wettability of the piezoelectric materials-HA [253].

### Interface reinforcement and nanotechnology

In general, biodegradable materials have suitable biocompatibility, including body compatibility and interface compatibility. When composite scaffolds are applied in tissue repair, the material-material and material-tissue interface compatibility plays an important role. In recent years, the development of interface reinforcement technologies has greatly improved the mechanical properties and biological properties (such as biological activity and tissue compatibility) of multicomponent-based scaffolds [254]. Among them, the modification of the scaffold surface by nanotechnology also plays an important role in preventing post-implantation infection and promoting bone tissue integration [255]. According to current research, interface reinforcement technologies can be roughly divided into three categories, including interface phase introduction (IPi), in situ growth (ISG) and surface modification (SM).

In composite scaffolds, a surface where two or more materials interact is called an interface in the composite material, which is not just a plane but a transitional area. The structure and properties of the material in this area are different from those of any one phase of the two or more materials, which is called the interface phase (IP). The IP is not only a link between the two phases of the materials but also a bridge for the transmission of stress and other signals [256]. In scaffolds made of composite materials, the common IPs include agents (such as zirconate, titanate and silane) and compatibilizers (such as poly(methyl methacrylate-co-methacrylic acid) and lysine triisocyanate) [257]. According to research reports, 3-(trimethoxysilyl) propyl methacrylate can enhance the compressive modulus of degradable composites (Mg/PCL) [258]. However, most of these compatibilizers and coupling agents are cytotoxic and may have an adverse effect on tissue repair. With abundant oxygen functional groups and surface negative charges, graphene oxide (GO) has become a good IP in composite scaffolds for bone defect repair in recent years. In order to solve the problem of poor bonding strength between biopolymers and bioceramics, Peng et al. [259] adopted SLS technology to introduce GO as the interface phase between biopolymer polyetheretherketone (PEEK) and HA, and prepared PEEK-HA/GO scaffolds, which has good biological activity and biocompatibility. At the same time, the compressive strength and modulus of PEEK-HA/GO scaffolds (65.41 MPa and 3.85 GPa) is significantly higher than that of PEEK-HAP (36.45 MPa and 2.71 GPa). ISG is an effective method for the preparation of composite materials; in ISG, two materials are combined by chemical bonding. Specifically, under the action of a nucleating agent, one material directly nucleates and grows on another material. Common nucleating agents are polydopamine, graphene and graphene oxide, which are mainly used to mineralize HA to prepare composite scaffolds [260]. In a recent study, graphene oxide was used as a nucleating agent to synthesize HA in situ on the surface of PLA to prepare PLA/HA@graphene oxide nanocomposites, showing significant cytocompatibility and high mechanical strength [261].

SM refers to a process for achieving new surface properties, such as hydrophilicity, biocompatibility and antistatic properties, while maintaining the original properties of materials or products. In recent years, with the good integration of scaffold materials and the improvement of interfacial interactions between materials in scaffolds, SM has been widely studied by researchers and is mainly achieved through physical and chemical methods, such as plasma spraying, flame spraying, microarc oxidation, laser ablation, sol-gel, surface grafting and electrodeposition [262]. In recent years, from the perspective of bone tissue development, anatomy and physiology, biomimetic SM technology has been favoured by a large number of scholars. According to the composition of bone (organic phase and inorganic phase), the SM of bone defect repair scaffolds is mainly achieved by the surface coating of similar biological components and materials [263]. Some nanostructured SMs have also been inspired by the microstructure of the bone surface [264]. Due to the similarity of the inorganic phase with bone, synthesized calcium phosphate (CaP) has become a common coating material, which is mainly applied by plasma spraying. After implantation, the ions released from the CaP coating on the implant surface will promote the formation of new bone tissue and combine with the coated implant [265]. However, some studies have pointed out that although a pure HA coating showed good osseointegration, it may affect the stability of early fixation after implantation [266]. The possible reason is that the pure HA coating has a higher crystallinity and low solubility, so there is a poor initial fixation. As an optimization method, it is a good choice to precisely control the more soluble amorphous constituents and select a more stable HA [267]. Due to its important role in maintaining the growth of bone cells and promoting the healing of damaged bone tissue, scaffolds are also often doped with Mg^2+^ to achieve improved performance [268]. Other metal ions can also be used as materials for coating scaffolds, such as strontium ions, silicon ions, fluoride ions, cobalt ions, superparamagnetic iron oxide nanoparticles and gold nanoparticles [149, 269]. It should be noted that before these materials are officially used in the clinic, long-term and rigorous experiments are required to observe whether the metal ions released upon scaffold degradation are toxic to cells or tissue.

Inspired by the organic phase of bone tissue, many biologically active proteins or cytokines are also used as coating materials, such as ECM-related proteins (such as collagen, integrins, chondroitin sulphate, and alkaline phosphatase (ALP)) [270, 271], cell-binding peptides (such as arginine-glycine-aspartic acid (RGD) and GFOGER) [272, 273] and growth factors (such as BMPs, PDGF, IGF I/II and TGF-β) [168, 274, 275]. However, the following points must be noted: 1) Proteins generally tend to adhere to the surface of high-surface-tension and nonpolar materials. During coating, external parameters should be considered, including the coating temperature, ionic strength and pH value [276]. 2) Only coating the surface of a material with peptides is usually insufficient to fully realize regeneration, and it is more appropriate to both modify the surface of the material and coat the surface with protein, which shows better performance in bone healing [270]. 3) Although many growth factors (such as BMP-2, BMP-7, PTH and PDGF) have been approved by the FDA for clinical use, inappropriate doses may cause adverse reactions, including osteolysis, unnecessary ectopic bone formation, cancer, and even death [52]. The porosity of the scaffold also affects the osteogenic induction that can be achieved with growth factors [277]. In addition, some growth factors (such as vascular endothelial growth factor (VEGF), fibroblast growth factor (FGF2, FGF9), placental growth factor (PGF) and BMPs) applied in a scaffold coating have been reported to promote bone repair and stimulate angiogenesis and vasculogenesis in the microenvironment of the local repair area [278].

In recent years, nanotechnology, comprising the study of the properties and applications of materials with structures ranging in size from 1 to 100 nm, has supplemented SM and represents the latest development in the field of SM [279]. When a material reaches the nanoscale, its performance will change suddenly, and special properties will appear. Nanotechnology can improve the biological function of materials by adjusting the surface parameters, mainly including the surface roughness, surface hydrophilicity, surface charge and surface nanotopography [219]. Surface nanoroughness is considered to promote osseointegration. Research indicates that differences in the surface nanoroughness of HA scaffolds can affect the osteogenic differentiation of BMSCs [280]. In general, surface hydrophilicity mainly regulates the adhesion and spreading of cells and can also improve the tissue healing process by regulating the state of immune cells [281]. Recently, D’Elía et al. [282] comprehensively evaluated the effects of the surface roughness and hydrophilicity of biodegradable materials, including nano-HA, on osseointegration, osteoconduction and osteoinduction. The surface charge of the scaffold contributes to the combination of ions and proteins after implantation and is essential for promoting cell attachment and growth [283].

Among surface parameters, the nanotopography (such as nanogrooves, nanopillars, nanotubes and nanodots) of the material has the most extensive effect on cells and the performance of scaffolds [284]. A recent study showed that a new type of biodegradable magnesium alloy (Mg-1.2%Nd-0.5%Y-0.5%Zr-0.4%Ca) modified by nanotechnology achieved a good balance between biodegradability and cytotoxicity [285]. Furthermore, the addition of HA nanoparticles to the PLA composite material significantly promoted protein adsorption and the spreading of murine calvarial preosteoblasts (MC3T3-E1) [286]. In addition, it has been reported that nanotopography can regulate the osteogenic differentiation of stem cells. Based on biodegradable materials, Xia et al. [287] evaluated the effects of HA bioceramic scaffolds with nanosheets, nanorods, and hybrid micro/nanorods on the proliferation and osteogenic differentiation of rat adipose-derived stem cells (ASCs). In vivo experiments in a rat skull defect model showed that nanotopography could significantly promote osteogenesis and angiogenesis. Some recent studies have shown that the surface of degradable nanofibrous biomaterials, such as nanofibrous gelatine, CS, PLA and PCL, can also affect the proliferation and differentiation of stem cells [288].

Nanotopography can also improve the mineralization and osseointegration of scaffolds. For example, it has been reported that a calcium phosphate coating with a 430 nm groove width can actively promote the surface mineralization of scaffolds [222]. Interestingly, the problem of infection after graft implantation can also be solved by improving the nanotopography [255, 289]. A recent study showed that 0.2% CS-coated calcium silicate-gelatine composite bone implants are more promising in bone defect repair than silver-coated implants [290]. Nanotopography can also help to create a good bone immune microenvironment, which is mainly achieved by regulating the attachment and spread of immune cells (such as macrophages, polymorphonuclear leukocytes and neutrophils) and changing the phenotype of macrophages [291].

## Intelligent materials and modular fabrication

The regeneration of bone tissue is realized in a series of complex microenvironments, which contain a series of environmental stimuli, including chemical conditions (such as pH, ionic strength and oxidation), physical conditions (such as temperature, electrical stimulation, magnetic fields and mechanical signals (stress/strain)) and biological signals (such as receptor-ligand recognition and enzymatic reactions). Intelligent material is a kind of special material which can make unique response to dynamic environment stimulation, especially in human body. Intelligent materials, usually polymers, biohybrid materials or cells, can “communicate” with the surrounding environment by integrating environmental stimuli and then responding (self-adjusting the state of the material). For example, p_11_–4, a type of self-assembling peptides, can be triggered by the body’s physiological pH to self-assemble after implantation, forming a self-supporting hydrogel in a concentration-dependent manner [292]. It should be noted that an intelligent material is usually unique and can show macroscopic functional behaviours in response to specific stimuli. To simultaneously respond to multiple stimuli in a complex microenvironment and perform complex functions, it is usually necessary to form an intelligent device by connecting multiple appropriate intelligent materials through modular manufacturing and assembly [18].

**Fig. 3:**
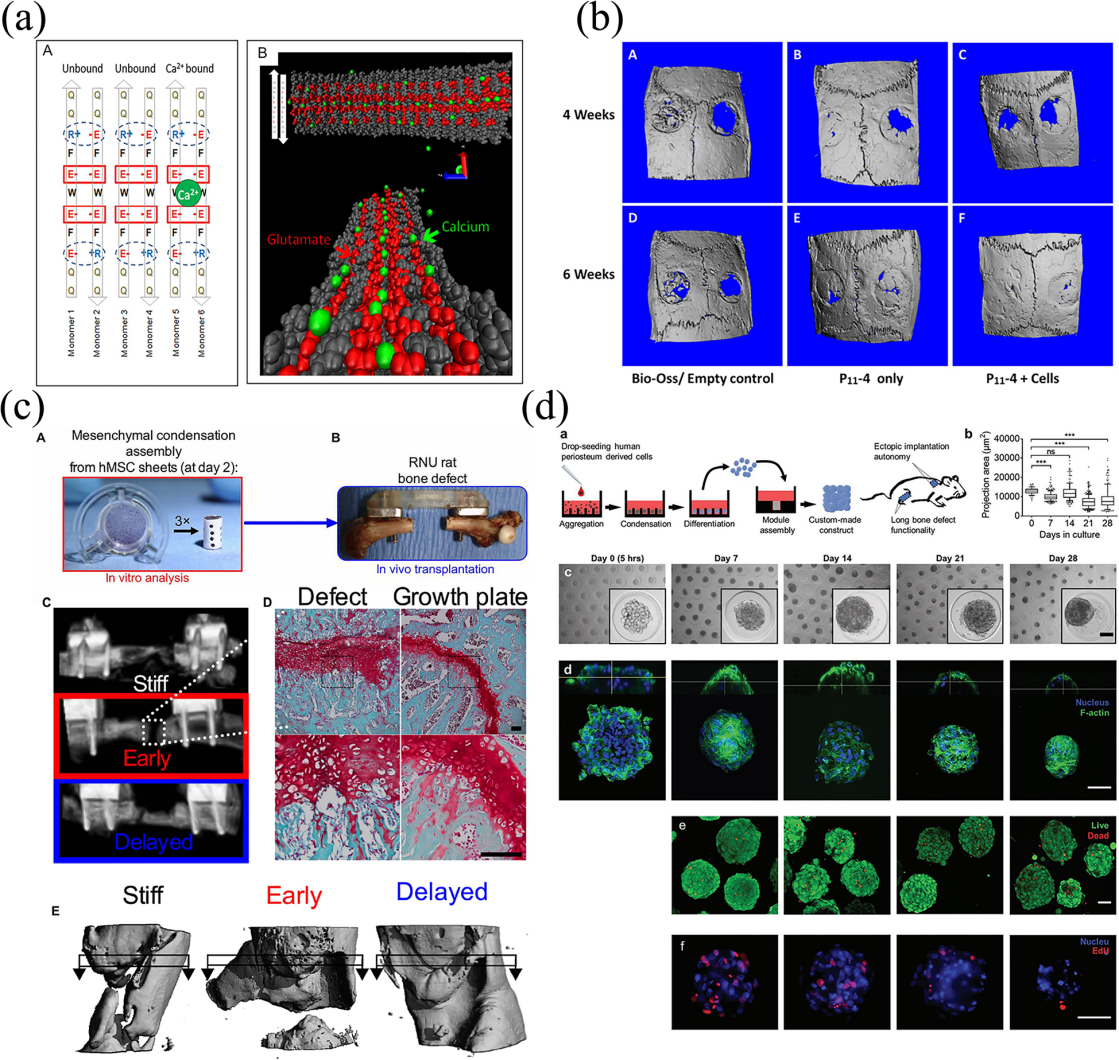
Representative intelligent materials and cell-based products for bone defect repair. **a** At a pH value of 7.4, the combination of the pH-sensitive, self-assembled β-peptide (SAP P11–4) and calcium ions in the AMBER software pair was siliconized. (a) The schematic shows the antiparallel arrangement of the 6 × P11–4 monomers and the preferred docking position of calcium (green) between 4 adjacent glutamic acid (E) residues (boxed, red). (b) Software simulation image shows the predicted arrangement of P11–4 bands related to calcium ions (green). **b** CT reconstruction of 4-mm 3D images of skull defects in rats of different groups. A and B are adapted by permission from [293], published by Elsevier. **c** The self-assembly of hMSCs to form bioactive tissues can be regulated by mechanical stress loading. (a) and (b) Schematic diagram of the repair of critical-size bone defects in rats with mesenchymal condensation assembly from hMSC sheets. (c) Representative in vivo micro-CT reconstructions at week 4 in each group (different mechanical stress loads). **d** Rats in the experimental group after the 4th week (left) were compared with rats in the control group, with a natural distal femur growth plate (right), by the saffron O/fast green staining of sagittal tissue sections. (E) Representative 3D micro-CT reconstructions at week 12 in each group. (c) is adapted with permission from [294], published by the American Association for the Advancement of Science. (c) Long-term culture of periosteal microspheroids. (a) Cell aggregation, differentiation, and modular self-assembly into a callus for repairing critical-size bone defects in mice. (c) Projection area of microspheroids over time (87–400 microspheroids). (c) Representative bright-field images of microspheroids over time. (d, e and f) F-actin, live/dead and proliferating cell (EdU) staining of microspheroids at different time points. Scale bars: c, d, e, f) 50 μm. D is adapted with permission from [24], published by Wiley

According to research, advanced bioactive scaffolds can possess a suitable porous structure, transfer growth factors, promote cell migration and proliferation and have suitable mechanical properties to cope with complex signals [295]. Some biodegradable materials (such as collagen, chitosan, fibrin, elastin, and hyaluronic acid) can promote cell adhesion through their natural adhesion ligands [296]. In addition, some studies have shown that many natural polymers, such as cellulose, chitosan and gelatine, have a lower critical solution temperature (LCST) phase transition and can respond to the change in temperature after implantation in the human body [297]. Iron oxide nanomaterials can sense and respond to the magnetic field in the microenvironment of tissue regeneration. Bock et al. dip-coated HA/collagen scaffolds with iron oxide nanoparticles to prepare a new type of magnetic scaffold that can support the adhesion and proliferation of human bone marrow stem cells [298]. Self-assembling structures based on peptides can also respond to microenvironmental signals, such as pH, ion concentration and temperature [299]. Recently, Saha et al. [293] prepared pH-sensitive, self-assembling *β*-peptides (SAP P_11_–4), which can reversibly switch between the liquid phase and the gel phase in response to pH changes in the microenvironment and is a new nucleating agent for HA (the simulated data from in silico modelling show that p_11_–4 fibres can form HA mineral nuclei through the negative charge region and attract calcium ions) (Fig. 3A). Next, in vivo experiments confirmed that when used to fill rat skull defects, P_11_–4 could significantly stimulate bone regeneration and promote bone defect repair (Fig. 3B). In addition, Sun et al. [300] prepared a new scaffold for repairing rabbit cartilage defects by combining self-assembled peptide nanofibres with decellularized cartilage matrix (DCM), which promoted the recruitment of endogenous MSCs at the defect site and played a positive role in cartilage and subchondral bone reconstruction.

Cell-based products are increasingly used in tissue engineering, some of which have predictable performance in vivo and support clinical transformation [23, 301]. Based on “developmental engineering” strategies, cell-based products can also be modularly fabricated [302]. Cells at an appropriate length scale can form temporary tissues by self-assembly, which can perform independent developmental procedures, thereby promoting organ formation [303]. Recently, Nilsson Hall et al. [24] developed a callus organism formed by the self-assembly of human periosteum-derived cells (hPDCs), which can be spatially bioassembled into multimodular constructs and used to repair critical-size bone defects in mice (Fig. 3D). In addition, human mesenchymal stem cells (hMSCs) can also form biologically active tissues by self-assembly and be regulated by mechanical stress loading to promote the repair of critical-sized (8 mm) bone defects in rats (Fig. 3C) [294].

## Conclusions and prospects

To date, the treatment of large bone defects remains a difficult problem that requires major investments in terms of medical costs, and the final therapeutic effect is not always satisfactory. Scaffolds made of biodegradable materials play an increasingly important role in the repair of bone defects. Of course, a clinically applicable scaffold needs to simultaneously possess the characteristics of biocompatibility, biodegradability, osteoconductivity, low immunogenicity and non-infectivity. Although many new materials have emerged with the development of science and technology, traditional biodegradable materials still maintain major advantages, including natural and synthetic degradable polymers, biodegradable ceramics and biodegradable metals, some of which have been approved for clinical application. For example, some natural biodegradable materials have natural adhesion ligands that can promote cell adhesion; synthetic biodegradable materials have excellent mechanical strength and can enhance cellular interactions; and biodegradable ceramics have good osteoconductivity and corrosion resistance. Although these materials also have shortcomings, they are still the cornerstone of efforts to develop a new generation of degradable materials.

To integrate the advantages of different materials, scaffolds made of composite materials are the current trend in bone defect repair. The latest technology to fabricate improved biodegradable materials has brought hope for the preparation of more biomimetic scaffolds for bone defect repairs, such as 3D and 4D printing, SM and nanotechnology. However, the biomimetic design of the scaffold should not be limited to the scaffold itself but should also include responses to various signals in the regeneration microenvironment. Therefore, intelligent devices (such as scaffolds) formed by the modular fabrication of intelligent materials represent the latest progress in the fourth generation of tissue repair scaffolds.

Generally, the treatment of bone defects is not a problem that can be solved by medicine alone but also require the joint efforts of molecular engineering, materials science, chemistry, mechanics and mathematics. For the use of biodegradable materials, in addition to long-term and rigorous experiments to verify their safety, it is also necessary to accurately control the degradation rate to match the rate of bone tissue regeneration and to provide suitable mechanical support for new bone tissue. We believe that these problems will be gradually solved with the development of intelligent materials and modular fabrication methods. It is a long and bumpy road for a new type of bone defect repair scaffold to successfully transition from the laboratory to the clinic, which requires the joint efforts of scientists and researchers in many fields. We hope this review can serve as a reference and provide some inspiration for researchers in related fields.

## Data Availability

The data and materials used during the current review are all available in this review.

## Abbreviations

ALP: Alkaline phosphatase
AM: Additively manufactured
CS: Chitosan
DCPA: Dicalcium phosphate anhydrous
DCPs: Dicalcium phosphates
DMP: Direct metal printing
EBB: Extrusion-based bioprinting
ECM: Extracellular matrix
EO: Endochondral ossification
FDA: US Food and Drug Administration
FDM: Fused deposition modeling
FGF: Fibroblast growth factor
GO: Graphene oxide
HA: Hydroxyapatite
hMSCs: Human mesenchymal stem cells
HYAFF-11: Benzyl ester of hyaluronic acid
IBB: Inkjet-based bioprinting
IMO: Intramembranous ossification
IP: Interface phase
IPi: Interface phase introduction
ISG: In situ growth
LAB: Laser-assisted bioprinting
LCST: Lower critical solution temperature
LIFT: Laser-induced forward transfer
MSc: Fibrin-mesenchymal stromal cell
OCN: Osteocalcin
PCL: Poly (ε-caprolactone)
PDS: Poly-para-dioxanone
PEEK: Biopolymer polyetheretherketone
PEO: Plasma electrolytic oxidation
PGA: Poly (glycolic acid)
PHB: Poly 3-hydroxybutyrate
PLA: Poly (lactic acid)
PLGA: Poly (lactic-co-glycolic acid)
PLLA: Poly (L-lactic acid)
Runx2: Runt-related transcription factor 2
S1P: Sphingosine 1-phosphate
SAON: Steroid associated osteonecrosis
SF: Silk fibre
Si-HA: Silicon-substituted hydroxyapatite
SLA: Stereolithography
SLS: Selective laser sintering
SM: Surface modification
TCP: Tricalcium phosphate
VEGF: Vascular endothelial growth factor
